# Palliative cardiovascular care: The right patient at the right time

**DOI:** 10.1002/clc.23307

**Published:** 2019-12-12

**Authors:** Mark F. Sullivan, James N. Kirkpatrick

**Affiliations:** ^1^ Inova Heart and Vascular Institute Falls Church Virginia; ^2^ Division of Cardiology University of Washington Medical Center Seattle Washington

**Keywords:** advance care planning, end of life, palliative care, symptom management

## Abstract

In the increasingly complex world of modern medicine, relationship‐centered, team‐based care is important in geriatric cardiology. Palliative cardiovascular care plays a central role in defining the scope and timing of medical therapies and in coordinating symptom‐targeted care in line with patient wishes, values, and preferences. Palliative care addresses advance care planning, symptom relief and caregiver/family support and seeks to ameliorate all forms of suffering, including physical, psychological, and spiritual. Although palliative care grew out of the hospice movement and has traditionally been associated with care at the end of life, the current model acknowledges that palliative care can be delivered concurrent with invasive, life‐prolonging interventions. As the population ages, patients with serious cardiovascular disease increasingly suffer from noncardiac, multimorbid conditions and become eligible for interventions that palliate symptoms but also prolong life. Management of implanted cardiac support devices at the end of life, whether rhythm management devices or mechanical circulatory support devices, can involve a host of complexities in decisions to deactivate, timing of deactivation and even the mechanics of deactivation. Studies on palliative care interventions have demonstrated clear improvements in quality of life and are more mixed on life prolongation and cost savings. There is and will remain a dearth of clinicians with specialist palliative care training. Therefore, cardiovascular clinicians have a role to play in provision of practical, “primary” palliative care.

## 1 INTRODUCTION

At any one time in the course of modern medicine, there are a host of ongoing advances in technology, physical therapy, nutrition science, and healthcare delivery. How can we evaluate which of these multifaceted modalities are right for the specific older patient suffering from cardiovascular disease within his/her clinical and social context? How can cardiovascular clinicians address the global needs of the increasing population of patients (and their caregivers) with multiple diseases? In the increasingly complex world of cardiovascular practice, relationship‐centered, team‐based care is sorely needed in geriatric cardiology. Palliative cardiovascular care plays a central role in defining the scope and timing of medical therapies and in coordinating symptom‐targeted care in line with patient wishes, values, and preferences.

## 2 HISTORY AND INITIAL CONCEPTION

“Palliative” comes from the Greek word “pallium,” a cloak‐like garment that was worn by the Greeks outside of common work‐life and was considered a form of protection. In the 15th century, English speakers modified the subsequent Latin word “palliatus” to form “palliate.” The term was advanced from the literal sense referring to the cloak one wears to the figurative means of protection and lessening the intensity of harm or disease.

The palliative care “movement” began in parallel to the hospice movement advanced by Dr. Cicely Saunders in mid‐20th century London and ultimately headquartered at St. Christopher's Hospice in 1967. In the United States, the idea of hospice care developed from a volunteer‐led movement. The first hospital‐based palliative care consult service was established in Detroit in the mid‐1980s with the first palliative medicine program opening its doors in 1987 in Cleveland, Ohio. While traditional palliative care was primarily focused on the care of patients suffering from oncologic diseases, it gained momentum in the care of patients with other ailments, especially those suffering from advanced heart failure. By 2005, it was estimated that close to 1.2 million patients received hospice care.

In 2004, palliative medicine received a nod from the Accreditation Council for Graduate Medical Education for the creation and funding of hospice and palliative medicine training programs. In 2006, members of the American Board of Medical Specialties voted to approve hospice and palliative medicine as a recognized specialty.

## 3 FROM DEFINITION TO CONCEPTUAL MODELS IN CARDIOVASCULAR MEDICINE

**Figure 1:**
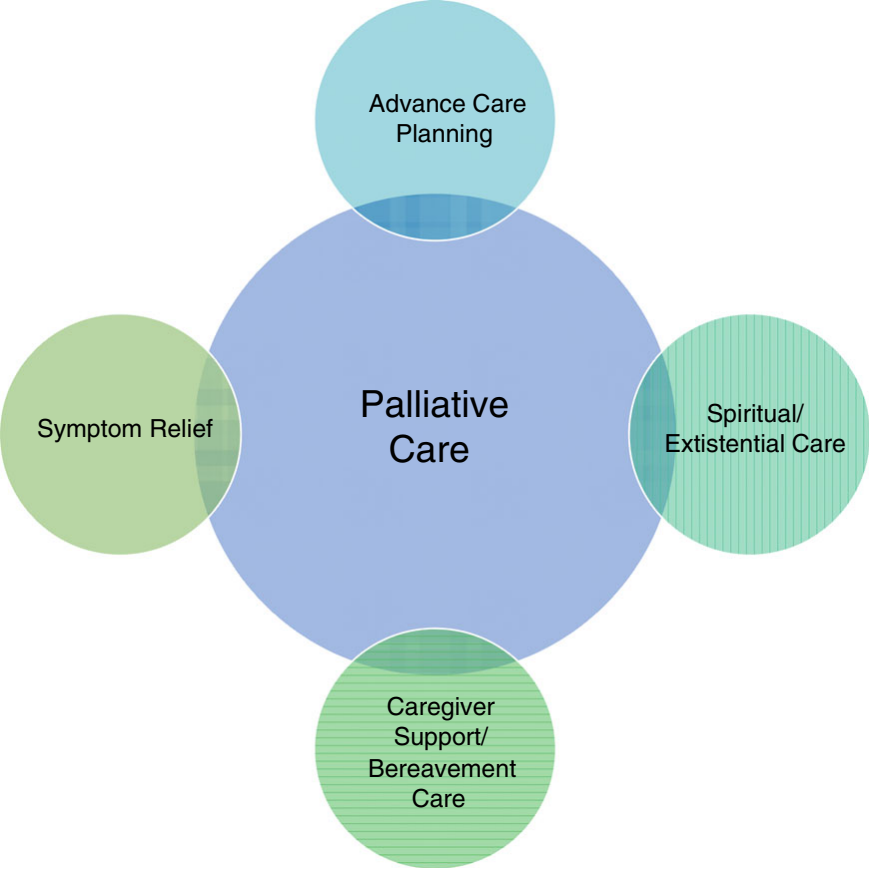
Elements of palliative care

Since the mid‐1990s, palliative care was recognized as a supportive approach to medical care that became considered by many to be appropriate for all patients with serious/life limiting disease. This response to the populations' increased triple burden of subacute, acute, and chronic disease was well demonstrated by the older and updated definitions of palliative care as proposed by the World Health Organization (WHO). In 1990, the WHO defined palliative care as “the active, total care of patients with progressive, far advanced disease and limited life expectancy whose disease is not responsive to curative treatment. It refers to the control of pain and other symptoms as well as treatment of social, psychological, and spiritual problems.”^1^ Nearly a decade later, the WHO updated its definition to “an approach that improves the quality of life of patients and their families facing the problems associated with life‐threatening illness, through the prevention and relief of suffering by means of early identification and impeccable assessment and treatment of pain and other problems, physical, psychosocial and spiritual”^2^ (Figure 1).

**Figure 2:**
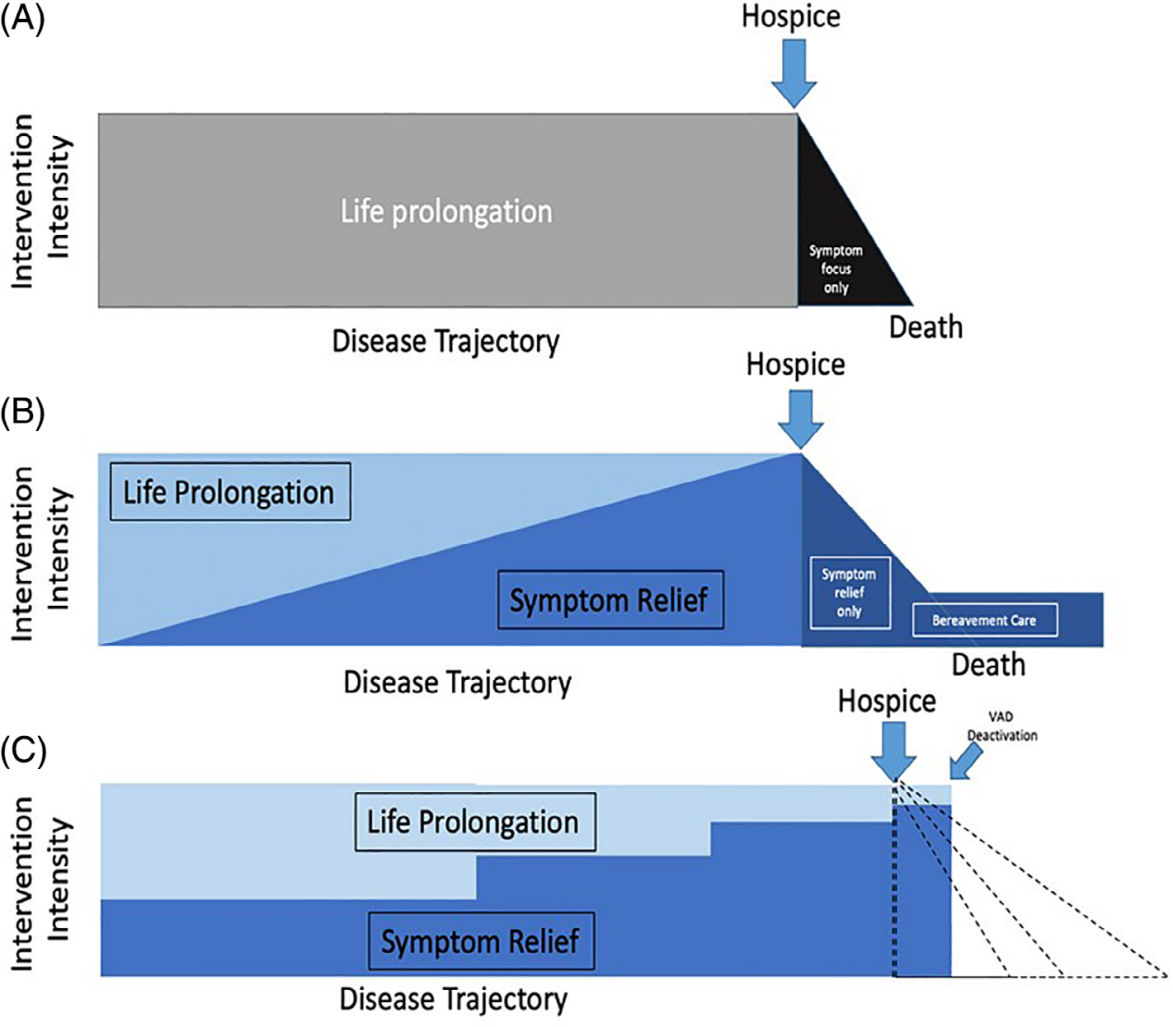
Conceptual models of palliative care. A, The “old” model of palliative care, featuring an emphasis on life prolongation until a stark transition point to hospice care. B, The current model of palliative care, in which life prolonging interventions and palliative care measures can coexist, with a greater emphasis on symptom relief and concurrent reduction in focus on life prolongation as the disease progresses toward death. The abrupt transition to hospice care remains. C, Proposed conceptual model to address some of the complexities of palliative cardiovascular care. At the point of hospice transition (though most patients with end stage cardiovascular disease do not enter hospice—hence the dashed line), many interventions for symptom relief that also prolong life are continued. If patients with mechanical circulatory support devices enter hospice, they usually do so with active devices but may have them deactivated later. For other patients with life‐limiting cardiovascular disease, the timing of death is difficult to predict, illustrated by the multiple dashed lines, particularly after deactivation of defibrillator therapy

The modification of the WHO definition parallels the evolution of medical practice in response to technological advancements, payment models, and health system policies that informed the practice of palliative care. In line with its origins, palliative care initially focused exclusively on the care of patients nearing the end of life (ie, synonymous with hospice). This former conceptual model advocated life prolonging interventions until these interventions were no longer effective, then the patient was turned over to the care of hospice providers, with a stark transition point (Figure 2A). Medicare/Medicaid funding for hospice services promoted (and still promotes) this conceptual model by requiring a <6 month prognosis for hospice entry and providing limited daily funding that often fails to cover symptom‐treating therapies like dobutamine.^3^

The contemporary conception of palliative care encompasses medical decision‐making, symptom relief, caregiver considerations, and restored quality of life on a much broader scale. There has been a recognition that the medical community must be better prepared to respond to all forms of suffering as important but distinct subjective, personal experiences that include pain, dyspnea, nausea, and so forth but also to feelings of guilt, depression, fear of dying, spiritual uncertainty, and a reflection of the attitudes of others.^4^ Moreover, all agree that compassionate responses to suffering must be personalized, conceived, and delivered in regard to patients' individual needs.^5^

This current conceptual model of palliative care recognizes the importance of palliative measures and a focus that is concurrent with life prolonging interventions, increasing in importance as life extensions become less possible and/or desirable. However, the stark transition at the point of hospice entry remains (Figure 2B). This concept of palliative care better reflects the clinical course for most patients with serious and life limiting diseases, but it fails to capture several important aspects of palliative cardiovascular care.

A potentially effective third model (Figure 2C) proposed by the authors takes into consideration many of the complicating factors of cardiovascular palliative care that are increasingly recognized and discussed in this review, while conserving the original intent of the aforementioned models. Within the complexities of cardiovascular care, changes in the focus on palliative measures can happen in more of a fluid fashion for patients with serious cardiovascular disease. Most cardiovascular therapies aim to prolong life, prevent catastrophe, and improve symptoms/quality of life. While the latter may become an exclusive focus later in the disease course, treatments such as revascularization, cardiac resynchronization therapy and mechanical circulatory support and heart transplant can produce different outcomes. They may be successful in reversing or stabilizing the pathophysiologic changes suffered by the patient; they may be rejected as inappropriate treatment options; or they may be attempted but fail to accomplish goals of care, with a variable degree and time course of success or failure (eg, initial complete or partial success that is not sustained).

At the point of hospice transition, many cardiovascular interventions for symptom relief that also prolong life are continued. If patients with mechanical circulatory support devices enter hospice, they usually do so with active devices but may have them deactivated later. Most would agree that decisions to deactivate or not replace devices should be accompanied by measures to provide palliation of symptoms that may follow (including psychological and existential distress). For many, if not most, patients with life‐limiting cardiovascular disease, the timing of death is difficult to predict, illustrated by the dashed lines in Figure 2C, even after deactivation of defibrillator therapy. In this proposed model, hospice entry remains a transition point away from initiating new life prolonging interventions, but some treatments should be continued in order to provide patient desired symptom relief and support patient defined quality of life.

## 4 PALLIATIVE CARE IN RELATION TO PROGNOSIS AND DECISION‐MAKING IN CARDIOVASCULAR MEDICINE

In the specific case of palliative care for older patients suffering from advanced cardiovascular diseases, clinicians practicing palliative care have often adapted approaches to care from the experience of patients with cancer. As cancer care has advanced, prognosis has become more difficult, but it has always been so in cardiovascular disease. Patients with heart failure, in particular, tend to experience periods of stability interrupted by episodes of severe symptoms that can progress suddenly to end stage. Before the widespread use of defibrillators, sudden arrhythmic death was a constant threat. Even after implantation of cardiac devices or heart transplant that can stabilize symptoms and prolong life, patients are still subject to undulations in symptoms.^6^ While clinical calculators have improved, the natural history of cardiovascular disease leaves prognostication elusive in many circumstances for particular patients. This is exacerbated by the complex pathophysiology of hypertension, diabetes, tobacco use, coronary artery disease, valvular disease, arrhythmia, and heart failure. The increasingly widespread adoption of device therapies, from implantable cardioverter defibrillators to mechanical circulatory support devices to percutaneous valvular interventions, has further complicated prognostication. [Correction added on 22 Jan 2020, after first online publication: In the previous sentence, “device” has been updated to “devices.”] In light of increasing prognostic uncertainty it has become more and more difficult to navigate the complex medications, nutritional regimens, operative and device‐based interventions, and rehabilitation programs that have responded to the evolution of medical care and illness.

Cardiovascular specialists provide expertise concerning interventions—their indications, risks, benefits, and limitations in helping patients to determine whether these interventions are right for them. Palliative care clinicians provide expertise in eliciting and exploring patients' hopes, fears, dreams, and anxieties in order to understand the patient's values, preferences, and goals. They also provide crucial expertise in palliating symptoms (especially noncardiac symptoms), whether or not patients opt for certain therapies.^7^ All of these elements are essential for helping patients make truly informed decisions regarding high stakes interventions.

Funding mechanisms that contribute to the delivery of healthcare have recently recognized the importance of specialist palliative care in end stage heart failure, to the extent that the Centers for Medicaid and Medicare Services now requires involvement of a palliative care clinician in support of advanced heart failure teams that care for patients with mechanical circulatory support devices.^8^ Other patients with serious and life limiting cardiovascular disease also face difficult decisions, serious symptoms and caregiver stress and may benefit from palliative care input. For example, some patients who are not candidates for destination left ventricular assist devices may be candidates for palliative home dobutamine. This decision can be a complicated one, as dobutamine is arrhythmogenic and may improve symptoms and allow patients to leave the hospital, but at the cost of earlier death. The development of catheter based, low risk alternatives to surgery is revolutionizing the treatment of severe valve disease, particularly in older patients. A recent study demonstrated that only a minority of older patients (mean age 84) seek transcatheter aortic valve interventions in order to prolong life, whereas palliation of symptoms and improvements in quality of life may play a bigger role.^9^, ^10^ This finding highlights the palliative nature of these devices for many older patients and the need for a holistic perspective in their care.

Additionally, an increasing number of older patients with serious noncardiovascular conditions are undergoing cardiac device implantation. Green et al demonstrated that patients older than 65 year with one or more serious noncardiac conditions have a high mortality rate 1 year after ICD implantation.^11^ Landes et al, recently reported on outcomes of oncology patients undergoing transcatheter aortic valve implantation. A review of LVAD recipients comparing 2008 to 2017 revealed a rise in the percentages of patients with cancer (3% increasing to 5%), severe diabetes (3%‐9.5%) and peripheral vascular disease (3%‐5%).^12^ Even after successful device implantation, these patients face life‐limiting or morbid conditions in the short term, as well as complicated questions about the interactions between their devices and other diseases.

## 5 PALLIATIVE CARE AT THE END OF LIFE FOR PATIENTS WITH CARDIOVASCULAR DISEASE

Palliative care is increasingly recognized to be important along the entire spectrum of disease for patients with serious and life‐limiting cardiovascular diseases. But it takes on a particular importance nearing the end of life. The European Society of Cardiology recommends end of life palliative care for patients with progressive functional decline, dependence in most activities of daily living, severe heart failure symptoms and poor quality of life despite optimized therapies, frequent hospital admission or other episodes of decompensated heart failure, noncandidacy for heart transplant or durable mechanical circulatory support, cardiac cachexia and clinical determination to be at end of life.^13^ Determining when to withhold or withdrawal cardiovascular interventions at the end of life, especially in relation to device therapy, can be a complicated endeavor, as can decisions about hospice. There is almost always “something else” that can be done to forestall death, whether a shock from an ICD, a transcatheter intervention or hemodynamic support from one of many mechanical circulatory support devices. Except in cases of end stage multi‐organ system failure, such interventions nearly always have some sort of physiological effect, but they may fail to meet goals of care, particularly in terms of meaningful life prolongation and the relief of suffering. These aspects likely play into the fact that hospice is underutilized in patients with heart failure,^14^ and may play a role in the finding that patients with heart failure spend little time in hospice prior to death.^15^

Palliative care plays a central role in determining when patient goals, values and preferences are inconsistent with device and other therapies or are consistent with hospice. Palliative care can, along with ethics consultation, resolve conflicts centered on withholding and withdrawing (or deactivating) devices. In addition, palliative care specialists can provide expert treatment of end of life symptoms that arise in the setting of device deactivation and/or facilitate referral to hospice and provide needed support to family and caregivers.

Several factors concerning modern death with cardiovascular disease complicate end of life decision‐making. There is a substantial variability in the timing of death following deactivation of many life sustaining cardiac devices. Unless patients with ICDs are in refractory ventricular fibrillation or life‐threatening ventricular tachycardia, timing of death can be highly variable. Timing of death in the setting of pacemaker and CRT deactivation can also be variable. A small study of terminally ill patients undergoing deactivation of ICDs did not demonstrate a significant difference in time to death between patients who were pacemaker dependent and those who were not.^16^ Another study found that a higher percentage of patients who had bradycardia therapies deactivated had died at 1 day and 1 week compared to patients with tachycardia therapies, but these differences were no longer significant by 1 month post deactivation.^17^

**Figure 3:**
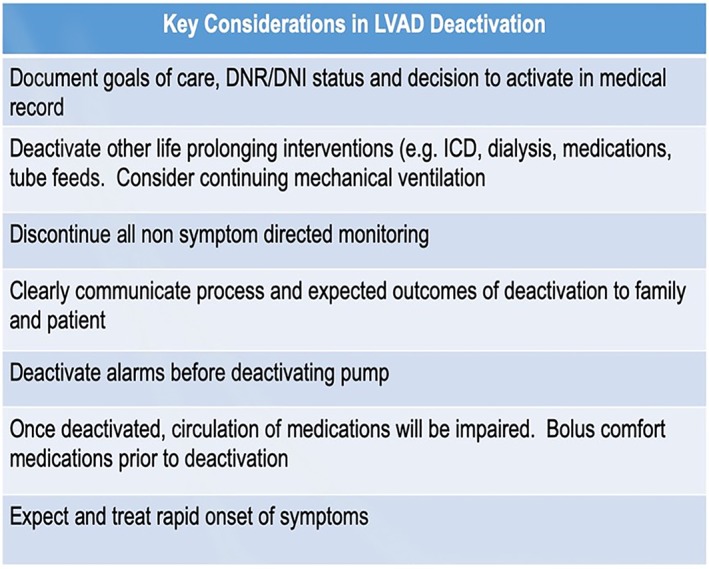
Key considerations in left ventricular assist device deactivation. Adapted from Gafford EF, Luckhardt AJ, Swetz KM. Deactivation of a left ventricular assist device at the end of life #269. J Palliat Med. 2013 Aug;16 (10):980‐2. doi: 10.1089/jpm.2013.9490. Epub 2013 Jun 14

Conversely, work by Dunlay and colleagues suggest that device deactivation precedes death for most patients with LVADs.^18^ Teuteberg demonstrated that a large majority of patients undergoing LVAD deactivation die within 1 hour.^19^ Work by Swetz and colleagues illustrate important decisions about LVAD deactivation that can be anticipated in “preparedness planning”—eliciting patient perspectives on LVAD deactivation in certain scenarios, including device failure, catastrophic complications of device therapy (such major stroke), inadequate quality of life and development of a debilitating comorbidity.^20^ Swetz and colleagues also prepared a checklist to guide deactivation (Figure 3).

Decisions about deactivation can be as complicated as deactivation itself. A number of studies have demonstrated heterogeneous perspectives on device deactivation among clinicians. Kramer et al demonstrated that physicians are generally comfortable with withdrawal of life sustaining interventions such as dialysis and ventilators but are far less comfortable deactivating ICDs and pacemakers.^21^ Daeschler et al reported that most electrophysiology clinicians did not see an ethical distinction between deactivating the shocking function of an ICD and foregoing external defibrillation but did see such a distinction in relation to deactivating pacemakers in a pacemaker dependent patient.^22^ Older studies have demonstrated that a significant percentage of patients may consider deactivation to be akin to physician assisted suicide or euthanasia and/or question the legality of deactivation.^23^ Not surprisingly, studies continue to demonstrate that a significant percentage of patients experience shocks after terminal diagnosis and within 1 month of death.

There are also heterogenous perspectives on deactivation of mechanical circulatory support devices. A study by McIlvennan et al highlighted differences in how LVAD deactivation is viewed between cardiovascular clinicians, who tended to view deactivation as a cause of death, as opposed to hospice and palliative medicine clinicians, who viewed the underlying disease process as the cause of death after deactivation. The cardiovascular clinicians indicated that deactivation was appropriate only after development of complications, malfunction or worsening other diseases, whereas the hospice and palliative medicine clinicians viewed deactivation as permissible whenever burdens of the devices were seen to outweigh benefits.^24^

## 6 PALLIATIVE CARE IN CARDIOLOGY: OUTCOMES

As evidence‐based cardiovascular science has advanced so too has evidence‐based evaluation of palliative medicine in the care of patients with cardiovascular disease in the inpatient, outpatient, and home‐based settings (Table S1).^25^ Most of the randomized controlled trials and observational studies examining the impact of palliative care on patient outcomes have focused on patients older than 67 years of age who could be candidates for advanced heart failure therapies. Based on predictive calculators, these patients are expected to have increased 6‐month to 1‐year mortality and face symptoms of fatigue, difficulty sleeping, shortness of breath, generalized weakness, nausea, sexual dysfunction, and bodily swelling. Researchers have examined primary and secondary outcomes including quality of life (as assessed by the validated Minnesota Living with Heart Failure and Kansas City Cardiomyopathy Questionnaires), symptom management, 30‐day hospital readmission, mood, spiritual wellbeing, mortality, caregiver understanding, and cost.

Sidebottom and colleagues examined patients with acute heart failure and randomized 116 patients to undergo multidisciplinary palliative care consultation that yielded a 3.06 point (95% CI: 2.75, 3.37) mean improvement in quality of life as measured by the Minnesota Living with Heart Failure Questionnaire. Patients in this study's treatment arm also reported a mean improvement in symptom burden of 4.31 points (95% CI: 4.00, 4.62) as measured by the Edmonton Symptom Assessment Scale.^26^ Rogers and colleagues studied a nurse‐practitioner led palliative care intervention in heart failure patients who has been hospitalized within the previous year and had an estimated 6‐month mortality risk of greater than 50%.^27^ The 75 patients who were randomized to the palliative care intervention also had a 9.5 point (95% CI: 0.94, 18.05; *P* = .03) reported improved quality of life as measured by the Kansas City Cardiomyopathy Questionnaire (KCCQ). Wong and colleagues developed a palliative transitional home nursing program that demonstrated a statistically significant improvement in quality of life and symptom burden. In addition, they found that the 41 patients with advanced cardiovascular disease in the intervention arm also had higher satisfaction with care at 4 weeks (48.84 points vs 3.55 points, *P* < .001) and a decreased hospital readmission rate (33.6% vs 61%, *P* = .009) at 12 weeks.^28^

Overall, there are mixed results in regard to the effect of palliative care interventions on mortality. Testing some of the components of palliative care have yielded positive results. Bekelman and colleagues randomized 187 out of 394 patients suffering from heart failure symptoms with self‐reported poor quality of life and limited functional status (KCCQ score < 60) to a multidisciplinary and collaborative disease management program vs standard of care. The intervention arm had decreased 1 year mortality (4.3% vs 9.67%, *P* = .04).^29^ Similar to other studies examining psychological outcomes, the authors also found a 2.1 point difference (95% CI, 0.43 to 3.78; *P* = .01) in self‐reported depression as measured by the validated PHQ‐9 questionnaire used to help guide treatment of anxiety and depressive symptoms. Evidence of improvement in mood was also replicated in the Sidebottom and Rogers studies. Rogers et al found patients in the intervention arm also experienced improved “spiritual wellbeing” at 6 months (mean difference 3.98 points, 95% CI: 0.46, 7.50; *P* = .027) as measured by the validated FACIT‐Sp tool.^27^ In a correlative study of 359 randomized patients employing destination therapy LVAD reading materials and patient video decision aids, Allen and colleagues found statistically significant correlations between patient‐stated values and caregiver‐reported treatment choices (difference in Kendall's tau: 0.36, *P* = .03). They also found decreased caregiver‐reported decisional conflict.^30^

## 7 WORKFORCE CHALLENGES IN CARDIC PALLIATIVE CARE

The rapid output of technologies and the dramatic growth of the older adult population will outpace the clinical and, probably, the funding infrastructure to support them. Relatively few workers now support over 57 million social‐security eligible beneficiaries, compared to a much more favorable ratio in the past.^31^ In light of the growing need for medical care for patients in older age brackets with cardiovascular disease who will face multimorbity and difficult decisions in an increasingly medically complex world, the need for palliative care will continue to expand. However, it is clear that there are not and will not be enough palliative medicine‐trained clinicians to meet the needs.

**Figure 4:**
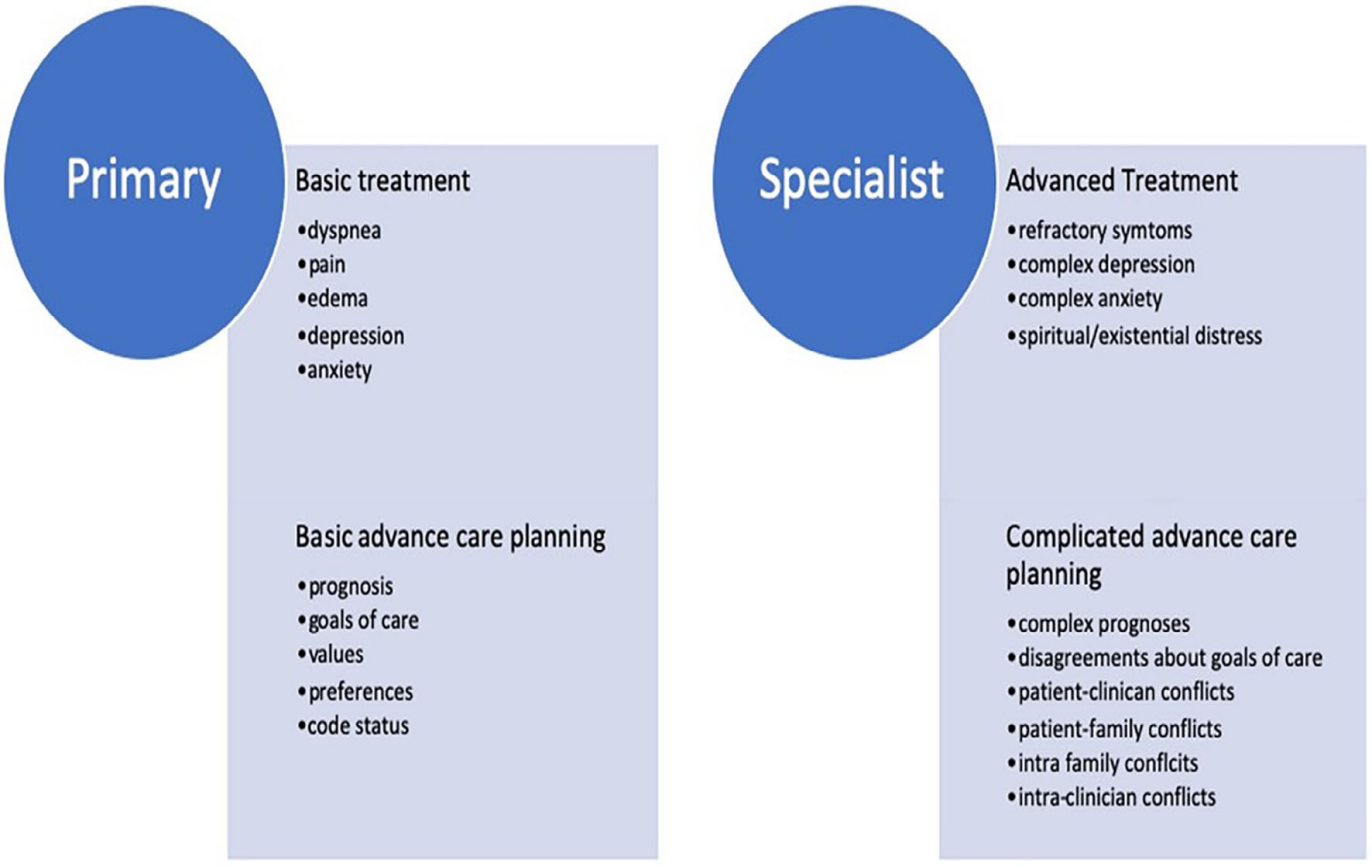
Important elements of primary vs specialty palliative care

**Figure 5:**
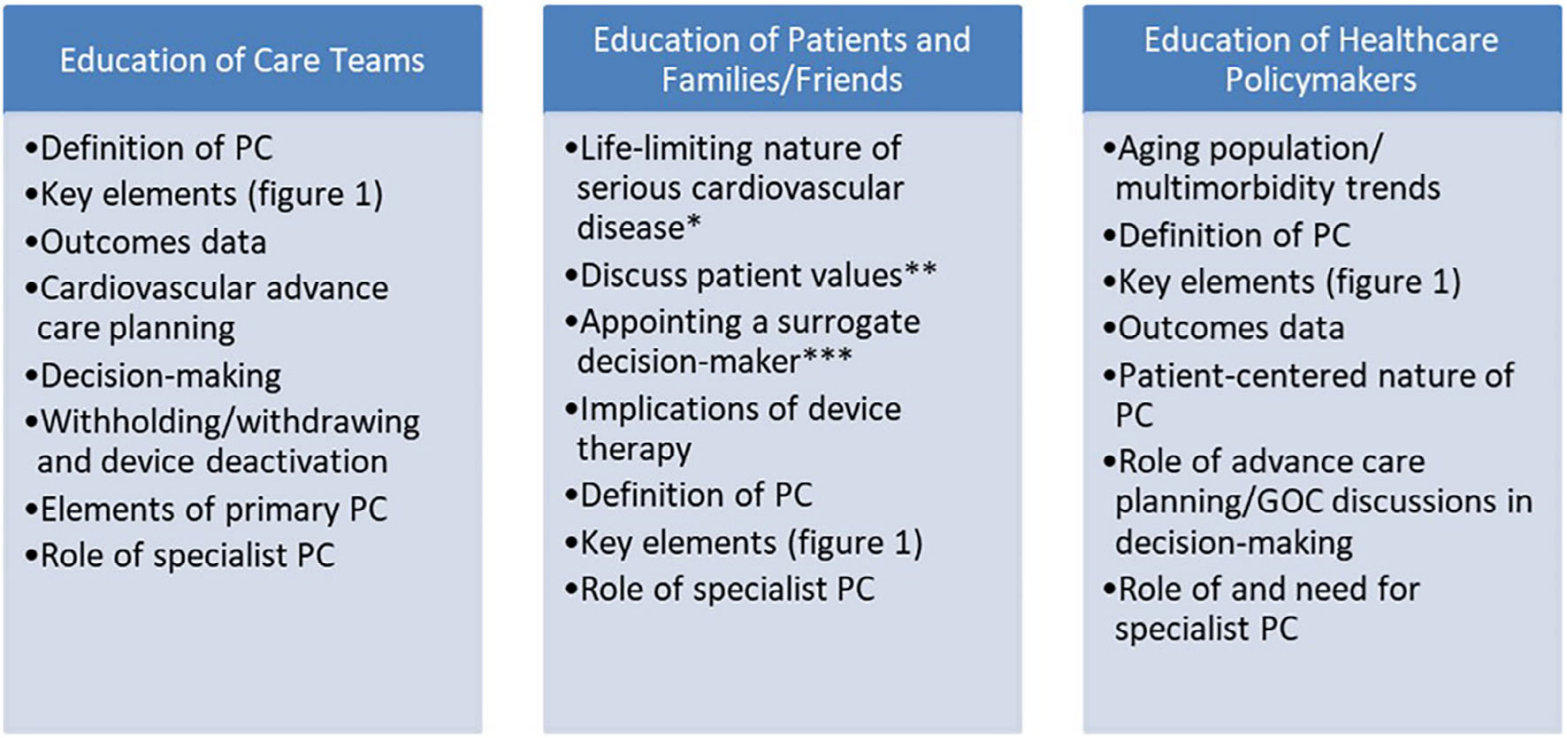
Educational approaches to facilitating palliative care in patients with cardiovascular disease. PC, palliative care; GOC, goals of care. *first area of emphasis in primary palliative care. **second area of emphasis in primary palliative care. ***third area of emphasis in primary palliative care

Clinicians without specialist training in palliative medicine must provide at least basic palliative care. A distinction has been made between “primary palliative care” and “specialist palliative care” to attempt to define what elements of palliative care can be handled by non‐specialists, and which require referral to clinicians with specialty training (Figure 4).^32^ These definitions will need to be refined and expanded in the cardiovascular context. Medical educators, especially in cardiology, must continue to explore ways in which to help the next generation of cardiovascular clinicians be comfortable and competent in addressing these challenges at the patient's bedside. Widespread adoption of primarily palliative care practices will require education of care teams patients and families/friends, and healthcare policy makers. Educational points of emphasis for each group are detailed in Figure 5.

## 8 PRACTICAL RECOMMENDATION FOR APPLYING PALLIATIVE CARE TO PATIENTS AND FAMILIES IN CARDIOVASCULAR MEDICINE

Cardiovascular clinicians providing primary palliative care are generally already expert in the treatment of cardiovascular symptoms. They will often need to partner with clinicians from other specialties to address noncardiac symptoms. The provision of primary palliative care also involves in understanding and communicating the nature of modern palliative care and in guiding patients in advance care planning and goals of care determination. It is essential to explain to patients and families that palliative care can coexist with life prolonging therapies, up to the point of transition to hospice care (and sometimes beyond). In the current complex hospital environment, it is easy for patients to be led to perceive that these are mutually exclusive. Clinicians should introduce the notion of palliative care and its components early on in the disease process, especially in relation to eventual device deactivation. This introduction can facilitate exploration of patients' values and goals of care (Table S2). Goals of Care discussions are important in many care settings, but particularly in the hospital. They may consist of simply reviewing advance care planning documents and code status but may require an extensive discussion in light of new diagnostic and therapeutic situations, particularly in relation to a refined prognosis. These goals of care discussion should inform shared decision‐making about life sustaining interventions and should include discussion of “what ifs” concerning when to withhold or withdrawal life sustaining interventions (such as ICD shocking function, mechanical circulatory support and valvular interventions).

The practical aspects of palliative care also include guiding patients to appoint one or more surrogate decision‐makers who can speak for the patient in the event the patient loses decision‐making capacity (Table S2). These surrogates must be made aware of the patient's preferences regarding interventions the patient would choose. A living will can help to guide surrogate decision‐makers in these instances, and there are many tools that can be used to facilitate communication of patient goals, values, and preferences to their surrogates. These tools include documents such as “The Five Wishes,” online questionnaires and even card games.^33^

Even with adequate preparation, decision‐making about withholding and withdrawing therapies, including device deactivation, can become complicated, involving strong emotions among friends and family. Clinicians should be prepared to address feelings, hopes and even conflict, and should seek help as needed from palliative medicine specialists, ethics consultants, and spiritual care providers.^34^

## 9 CONCLUSIONS

The practice of palliative care has evolved over time, but the fundamentals of advance care planning, symptom treatment and caregiver support through a relationship‐centered, holistic, team‐based approach remain. Evidence‐based trials and observational studies have demonstrated that a palliative care approach in support of the care of the patient can relieve suffering and facilitate better communication regarding what matters most to individuals facing the realities of morbidity, suffering, and death. [Correction added on 22 Jan 2020, after first online publication: In the previous sentence, “relief” has been updated to “relieve.”] To geriatric patients with serious cardiovascular disease, palliative care offers a holistic, multidisciplinary and evidence‐based approach, alongside cardiac interventions, to improve quality of life.

## CONFLICT OF INTEREST

The authors report no conflicts of interest related to this project.

## Supporting information


**Appendix S1.** Supporting Information
