# Supplementary material for: Palliative cardiovascular care: The right patient at the right time
Source: Clin Cardiol. 2019 Dec 12;43(2):205–12. doi: 10.1002/clc.23307 (PMC7021658; doi:10.1002/clc.23307)
Supplement: Supplementary file 1 — Appendix S1. Supporting Information [file CLC-43-205-s001.pdf]

Supplementary Table 1

Representative Trials: Palliative Care in Heart Failure					
	**Authors**	**Patient Population**	**Intervention (Participants Randomized)**	**Control (Participants Randomized)**	**Results**
**Inpatient Specialty Palliative Care**	Hopp et al, 2016	Acute HF, 1-yr mortality risk of ≥33%, and/or NYHA Class III–IV (Mean age: 68)	Inpatient specialist consultation from a multidisciplinary team (physician, nurse practitioner, chaplain, social worker) conducted clinical interview(s), assessing symptoms, goals of care and post-treatment location desires, and advance care planning (n=43)	Usual care (n=42)	**Hospice utilization/ACP** (Composite outcome): NS; difference between groups 9.3% (95% CI: −11.8%, 30.0%)
	Sidebottom et al, 2014	Acute HF (Mean age: 73)	Specialty multidisciplinary palliative care consultation assessing physical and emotional symptoms, spiritual, and social aspects of care. (n=116)	Usual care (n=116)	**QOL** [Minnesota Living with Heart Failure Questionnaire]: Improved, mean difference 3.06 points (95% CI: 2.75, 3.37)<br><br>**Symptom burden** [ESAS]: Improved total symptom burden, mean difference 4.31 points (95% CI: 4.00, 4.62)<br><br>**Six-month mortality:** NS; HR, 1.90 (95% CI: 0.88, 4.09)<br><br>**30-day hospital readmission**: NS; HR, 1.43 (95% CI: 0.5, 4.1)<br><br>**Hospice use within 6 months**: NS; HR, 1.60 (95% CI: 0.58, 4.38)<br><br>**ACP within 6 months:** Improved; HR, 2.87 (95% CI: 1.09, 7.59)<br><br>**Mood** [PHQ-9]: Improved; mean difference, 0.72 (95% CI: 0.41, 1.03)

**Outpatient Specialty Palliative Care**	Rogers et al, 2017	Hospitalization for HF in past year and ESCAPE score >=4 indicating >50% risk of 6-mo. mortality. (Mean age: 71)	Interdisciplinary NP-led specialty palliative care intervention concomitant with usual HF management. Intervention foci included: physical and emotional symptom management, spiritual concerns, and advance care planning. (n=75)	Usual care (n=75)	**QOL** [KCCQ]: Improved at 6 months, mean difference 9.49 points (95% CI: 0.94, 18.05; p=0.03)<br>[FACIT-Pal]: Improved at 6 months, mean difference 11.77 points (95% CI: 0.84, 22.71; p=0.035)<br><br>**Mood** [HADS depression]: Improved at 6 months, mean difference −1.94 points (95% CI: 3.57, −0.31; p=0.02)<br>[HADS anxiety]: Improved at 6 months, mean difference −1.83 points (95% CI: −3.64, −0.02; p=0.048)<br><br>**Spiritual wellbeing** [FACIT-Sp]: Improved @ 6 months, mean difference 3.98 points (95% CI: 0.46, 7.50; p=0.027)<br><br>**6-month mortality:** NS, 30.7% vs 26.7% (p value not reported)<br><br>**HF-related Rehospitalization:** NS, 30.7% vs. 29.3% (p value not reported)

**Home-Based Specialty Palliative Care**	Allen et al, 2018	NYHA Class III-IV HF	Approach comprised of clinician education, use of DT LVAD pamphlet and patient video decision aids (n = 248)	Usual care (n = 111)	**Caregiver knowledge:** Improved 64.2 % to 73.7 % vs. 62.6% to 76.4% +/- 4.8% (p=0.08)<br><br>**Patient stated values correlation to caregiver-reported treatment choice:** Difference in Kendall's tau: 0.36 (95% CI: 0.04 to 0.71; p=0.03)<br><br>**Caregivers reported decisional conflict (0 to 100) at baseline:** <u>Control group</u>: 19.0 ± 2.1<br><u>Intervention group</u>: 21.4 ± 2.6, which decreased post-education more in the control group<br><u>Control group</u>: 9.0 ± 1.9<br><u>Intervention group</u>: 18.8 ± 2.4 (p=0.009)
	Brännström et al, 2014	NYHA Class III–IV HF (Mean age: 79)	Predominately in-home HF disease management and palliative care services via a multidisciplinary approach and care coordination (n=36)	Usual care (n=36)	**QOL** [EQ5D]: Improved; (57.6 ± 19.2 vs. 48.5 ± 24.4 (p=0.05) [KCCQ]: NS (data not reported)<br><br>**Symptom burden** [ESAS]: NS (data not reported)<br><br>**Six-month survival**: NS; (p=0.34)<br><br>**Hospitalizations**: Reduced; mean (SD)=0.42 (0.60) vs. 1.47 (1.81); p=0.009<br><br>**Total costs:** NS; Mean €4078 vs. €5727 (p not reported)<br><br>**Increased proportion of patients with improved NYHA class:** 39% vs. 9% (p=0.015)
	Wong et al, 2016	Advanced HF (e.g. NYHA stage III–IV) (Mean age: 78)	Palliative care home nurses conducted home visits/telephone calls providing transitional palliative care (n=43)	Usual care (n=41)	**Symptom burden** [ESAS]: Proportion of patients with improvement in total score, 73% vs. 41.4% (p<0.05)<br><br>**QOL** [McGill]: Improved at 4 weeks; 7.57 points vs. 6.46 points (p<0.001)<br>[Chronic HF Questionnaire]: Improved at 4 weeks; 5.26 points vs. 4.47 points (p<0.001)<br><br>**Satisfaction with care**: Higher at 4 weeks; 48.84 points vs. 3.55 points (p<0.001)<br><br>**Hospital readmission**: NS at 4 weeks; 20.9% vs. 29.3% (p=0.38); reduced at 12 weeks: 33.6% vs. 61% (p=0.009)

**Primary Palliative Care/ Collaborative Care Models**	Bekelman et al, 2015	HF with poor QOL, limited functional status, and significant symptoms (KCCQ score <60) (Mean age: 68)	Multidisciplinary collaborative HF disease management, and tele- monitoring with patient self-care support (n=187)	Usual care (n=197)	**QOL** [KCCQ]: NS at 1 year; 54.2 (95% CI: 51.7, 56.6) vs. 53.6 (95% CI: 51.1, 56.0)<br><br>**Mortality:** Decreased at 1 year; 4.3% vs. 9.67% (p=0.04)<br><br>**Mood** [PHQ- 9]: Improvement in depression among patients with initial positive screen; mean difference, 2.1-point reduction (95% CI: 0.43, 3.78; p=0.01)<br><br>**Hospital readmission**: NS at 1 year; 29.4% vs. 29.9% (p=0.87)

Representative trials examining palliative care in heart failure

Adapted from Kavalieratos D, Gelfman LP, Tycon LE, et al. Palliative care in heart failure: rationale, evidence, and future priorities. *J Am Coll Cardiology*. 2017 Oct 10;70(15):1919-1930. doi: 10.1016/j.jacc.2017.08.036

Supplementary Table 2

Practical Aspects of Advance Care Planning in Primary Palliative Care
**Introducing the nature of cardiovascular disease**
• "While modern cardiovascular treatments can go a long way to give patients a good quality of life and extend how long they can live, we know that many cardiovascular conditions like yours ultimately limit a person's lifespan and can prolong life when quality of life is not good."
• "Many patients with serious cardiovascular diseases will face difficult decisions at the end of life when considering life prolonging therapies."
**Exploring patient values**
• "Tell me about the things that make life meaningful for you."
• "What do you hope for?"
• "What are you most concerned about right now/in the future?"
• "There are some people who could want to go on living and would want care focused on comfort if they could not do certain things (like participate in certain activities or have meaningful interaction with loved ones), and other who would want to have life prolonged at all costs. How would you answer for yourself?"
**Identifying a surrogate decision-maker**
• "If you could no longer make medical decisions for yourself, who do you think would be the person who knows your wishes the best?"
• "Who would be best the best person to represent your wishes?"
• "Have you told this person about your wishes and that you would choose her or him to be your decision-maker?"
• "Have you ever discussed with this individual what is important to you, what goals you have and what medical treatments you would want or not want in an end of life situation?"

Adapted from 2019 ACC Expert Consensus Decision Pathway on Risk Assessment, Management, and Clinical Trajectory of Patients Hospitalized With Heart Failure: A Report of the American College of Cardiology Solution Set Oversight Committee. *J Am Coll Cardiology* 74(15): 1966-2011.
